# ‘I Think Smoking’s the Same, but the Toys Have Changed.’ Understanding Facilitators of E-Cigarette Use among Air Force Personnel

**Published:** 2020-08

**Authors:** MA Little, K Pebley, K Porter, GW Talcott, RA Krukowski

**Affiliations:** 1 University of Virginia, School of Medicine, Department of Public Health Sciences, 560 Ray C. Hunt Drive, Charlottesville, VA, USA; 2 University of Memphis, Department of Psychology, 400 Innovation Drive, Memphis, TN, USA, 38152; 3 Wilford Hall Ambulatory Surgical Center, 59 MDW/ 59 SGOWMP, 1100 Wilford Hall Loop, Bldg 4554, Joint Base Lackland AFB, TX, USA 78236; 4 Department of Preventive Medicine, University of Tennessee Health Science Center, 66 North Pauline Street, Memphis, TN, USA 38163

**Keywords:** E-cigarettes, Vaping, Electronic cigarettes, Military, Young adults, Designated tobacco use areas, Policy

## Abstract

**Background:**

The military has stringent anti-tobacco regulations for new recruits. While most tobacco products have declined in recent years, e-cigarette use has tripled among this population. However, little is known about the factors facilitating this inverse relationship.

**Objectives:**

Examine the facilitators of e-cigarette use during a high risk period following initial enlistment among young adults.

**Methods:**

Focus groups were conducted with Airmen, Military Training Leaders (MTLs) and Technical Training Instructors (TTIs) to qualitatively explore unique characteristics of e-cigarettes leading to use in Technical Training.

**Results:**

The most commonly used tobacco product across participants was cigarettes (42.7%), followed by e-cigarettes (28.0%) and smokeless tobacco (22.6%). Almost a third (28.7%) of participants reported using more than one tobacco product. E-cigarette use was much more common among Airmen (76.1%), compared to MTLs (10.9%) and TTIs (13.0%).

Four main facilitators around e-cigarette use were identified including: 1) There is no reason not to use e-cigarettes; 2) Using e-cigarettes helps with emotion management; 3) Vaping is a way of fitting in; and 4) Existing tobacco control policies don’t work for vaping. E-cigarettes were not perceived as harmful to self and others, which could explain why Airmen were much less likely to adhere to existing tobacco control regulations. Subversion was viewed as the healthy option compared to utilizing designated tobacco use areas due to the potential exposure to traditional tobacco smoke. This coupled with a lack of understanding about e-cigarette regulations and difficulties with enforcement, promoted use among this young adult population.

**Conclusion:**

Findings suggest that e-cigarettes are used for similar reasons as traditional tobacco products, but their unique ability to be concealed promotes their widespread use and circumvents existing tobacco control policies. In order to see reductions in use, environmental policies may need to be paired with behavioral interventions at the personal and interpersonal level.

## Introduction

With the introduction of electronic cigarettes (e-cigarettes) to the market in 2006, there has been a dramatic increase in their use. A recent nationally-representative survey in the United States (U.S.) indicates that past 30-day e-cigarette use among 18-24 year olds has increased significantly from 2.4% in 2013 to 7.6% in 2018 [1,2]. Similarly, during that same time period e-cigarette use increased from 5.4% to 15.3% among 43,597 newly enlisted Airmen (called Airmen regardless of gender or rank) surveyed about their tobacco use prior to enlistment [3].

Although some of the increase in e-cigarette use is based on their perceived safety over conventional cigarettes [4], much is still unknown about the health risks associated with using e-cigarettes. Recent literature suggests that e-cigarettes may place users at increased risk for lung disease due to exposure to high levels of ultrafine particles and other toxins [5-7]. Additionally, e-cigarette use is associated with increased use of other tobacco products one year later among recently enlisted Airmen. Klesges et al. found that current e-cigarette-only users at baseline were 6.4 times as likely to convert to conventional cigarette use and 10.1 times as likely to convert to non-cigarette tobacco product use (e.g., smokeless tobacco, hookah, cigars) at a one year follow-up when compared to never-users [3].

Given the conversion rate between e-cigarettes and traditional forms of tobacco (e.g., cigarettes and smokeless tobacco), the health impact of traditional tobacco, and the probable health effects from e-cigarette use alone [5], the growing prevalence of e-cigarette use among young adults may increase health risks while leading to significant financial costs, particularly for young adults entering the military. For instance, the Department of Defense (DoD) spends on average $1.6 billion annually treating tobacco-related morbidity among active duty military personnel (e.g., medical care, hospitalizations, lost work days) [8].

Although the military has taken steps to reduce tobacco use over the past several decades, traditional policies may be limited in their effectiveness for non-cigarette tobacco products, such as e-cigarettes. Airmen have an enforced abstinence period during Basic Military Training (8 ½ weeks) and the first four weeks of Technical Training which appears to reduce cigarette smoking by 18% among Airmen who reported smoking prior to joining the Air Force [9]. Furthermore, Airmen are not allowed to use tobacco during the duty day (between breakfast and dinner) throughout Technical Training [10], which can last up to 18 months; however, the effectiveness of this policy is unclear. A previous review of the literature found that while interventions may reduce the rate of illegal sales to youth initially, lack of enforcement and the ability for youth to acquire tobacco from social sources may undermine their effectiveness [11]. In the Air Force, the majority of former users of traditional tobacco products re-initiate after the ban is lifted and many non-users start using tobacco for the first time in Technical Training [12,13]. While even less is known about the effectiveness of these policies with regard to e-cigarette use, this high rate of (re)initiation suggest that other factors, such as peer influences or enforcement issues may limit the effectiveness of these environmental interventions.

In October 2019, the DoD removed e-cigarettes from its shelves [14]. Unfortunately, research suggests that Airmen do not primarily purchase e-cigarettes on base, limiting the potential effectiveness of an on-base availability policy for e-cigarettes [15]. Targeted marketing and price promotions for military personnel may further reduce the effectiveness of DoD policies. For instance, JUUL, a leading e-cigarette company, recently launched the heroes.juul.com website, which is devoted to targeted marketing (e.g., promotional videos featuring active duty and veterans) and price promotions (e.g., $1 dollar JUUL starter kits) for military personnel and veterans [16]. Therefore, it may be necessary to fully explore the environmental facilitators of e-cigarette use, as the most promising solutions to decrease use may be different from the traditional toolbox. Given the high prevalence of e-cigarette use among new recruits entering the military, understanding the facilitators of e-cigarette use in the military population may be an important bellwether for other vulnerable populations (e.g., non-college attending young adults).

Thus, the current study sought to identify facilitators of e-cigarette use at a personal (e.g., individual-level characteristics, beliefs, and skills related to tobacco use), interpersonal (e.g., friend and social network influences), and environmental level (e.g., cultural values, norms and the built environment) among Airmen undergoing Air Force Technical Training. Given that most of the tobacco use (re)initiation has traditionally occurred during Technical Training [12,13,17], it is important to understand what factors are influencing Airmen to use e-cigarettes during this high risk time, particularly since they are increasing in popularity and existing tobacco regulations may be limited in effectiveness. These findings will inform intervention and policy efforts for youth and young adults who have similarly experienced a rise in e-cigarette use due to targeted marketing and price promotions [18].

## Materials and Methods

This study is a qualitative exploration of the tobacco experience of Airmen in Technical Training (referred to as Airmen for the remainder of the manuscript), Military Training Leaders (MTLs) and Technical Training Instructors (TTIs). Data were collected as part of a larger study exploring factors predicting tobacco use among Airmen during Technical Training. This paper presents findings relevant to personal, interpersonal, and environmental facilitators of e-cigarette use. In this study, we discuss the results of focus groups with Airmen undergoing Air Force Technical Training, as well as focus groups of MTLs and TTIs across the five largest Technical Training schools where the majority of non-prior service Airmen are trained. Study procedures were approved by the 59^th^ Medical Wing Institutional Review Board.

### Participants and recruitment

We conducted a total of 22 focus groups (N=164 participants) among Airmen (n=10), MTLs (n=7), and TTIs (n=5) from July 2018 to February 2019 at Joint Base San Antonio - Fort Sam Houston and Lackland Air Force Base (AFB), Goodfellow AFB, and Sheppard AFB in Texas and Keesler AFB in Mississippi. MTLs are the direct supervisors of Airmen, ensuring they are where they are supposed to be and dispensing disciplinary action. TTIs are responsible for teaching the specific skills required for that career field. While MTLs are always active duty, TTIs can be active duty or civilians (typically Airmen who have separated or retired from the military). For this study, Airmen volunteers were recruited during their out-processing week at the end of their Technical Training when they were receiving health-related briefings. MTL and TTI volunteers were recruited through a recruitment email sent by the Senior MTL at each base. Participants had to be at least 18 years of age and could be either a tobacco or non-tobacco user.

### Focus group procedures

Protocols for the focus group were developed for users and non-users as well as MTLs and TTIs. The focus group questions targeted the following domains: (1) personal experience with tobacco, (2) facilitators of tobacco use on base, (3) barriers to tobacco use on base, and (4) strategies to reduce tobacco use among Technical Trainees. This paper focuses specifically on data related to e-cigarette use.

Focus groups were conducted by two of five trained non-military researchers in a private room without leadership present in order to promote an open and safe environment. Each focus group contained one moderator and at least one note-taker. Participants were provided with an informational consent letter and verbally consented to participate. Focus groups contained, on average, 7 participants, ranging from 4 to 11 participants, and took on average 45 minutes to complete. Airmen focus groups were separated by tobacco use status (n=7 with tobacco users, n=2 with non-users, n=1 with users and non-users). All MTL and TTI focus groups were mixed with tobacco users and non-users. Participants were provided with food during the focus group. Responses were anonymous and audio-recorded.

### Analysis

Transcripts of focus groups were transcribed by Datagain. Transcripts were checked by researchers before coding. A hybrid deductive-inductive approach was used to code transcripts. Two trained research staff members coded each transcript. Coders met to resolve discrepancies and came to agreement. If an agreement could not be reached, a third coder was brought in to resolve discrepancies. The research team used *NVivo* (v12) software to manage the coding process.

To facilitate the first pass of coding, a codebook was developed using the social ecological model, overarching research questions from the grant, and evidence from the literature. As mentioned above, the primary domains within this initial codebook were facilitators and barriers of tobacco use during training, personal experiences with tobacco and strategies to reduce tobacco use among trainees. Initial codes within domains were identified based on the literature. Next, researchers reviewed meaning units within each discrete code to ensure adherence to operational definitions and to determine whether codes should be merged or sub-coded. Additionally, meaning units within codes were categorized based on the specific tobacco product mentioned, including e-cigarettes. Finally, individual codes were organized into larger categories.

## Results

Of the 164 participants, 83 (50.6%) were Airmen, 48 (29.3%) were MTLs and 33 (20.1%) were TTIs. The majority of participants were male (77.4%) and tobacco users (72.0%). The most commonly used tobacco product across all participants was cigarettes (42.7%), followed by e-cigarettes (28.0%) and smokeless tobacco (22.6%). Almost a third (28.7%) of participants reported using more than one tobacco product. E-cigarette use was much more common among Airmen (76.1%), compared to MTLs (10.9%) and TTIs (13.0%).

Airmen, MTLs and TTIs were asked what about the Technical Training environment facilitates tobacco use. Four main facilitators around e-cigarette use were identified including: 1) there is no reason not to use e-cigarettes; 2) using e-cigarettes helps with emotion management; 3) vaping is a way of fitting in; and 4) existing tobacco control policies don’t work for vaping. Facilitators are described in detail in the following sections and supported by quotations from Airmen, MTL and TTI focus group participants in Tables 1-4.

### No reasons not to use e-cigarettes

A number of reasons emerged under the idea that there is no reason not to use e-cigarettes (Table 1). Specifically, Airmen, MTLs and TTIs mentioned that e-cigarettes had many positive attributes, were not perceived as harmful, were cool gadgets and were seen as easy to use.

Airmen, MTLs and TTIs all discussed the perception that e-cigarettes are not (perceived as) harmful. One Airmen said, ‘Vaping is hardly tobacco.’ A MTL thought that Airmen don’t realize the harms of e-cigarettes, and think ‘this smells like bubble gum so I’m just gonna do it.’ A TTI said, ‘it doesn’t smell and you can get the same nicotine, but it has got this flavor and it doesn’t taste like a normal cigarette. You can get chocolate or strawberry or bubble gum.’ E-cigarettes were also seen as not harmful in terms of time required; a TTI said, ‘You just hit it once and you are done…Instead of lighting up a cigarette and letting it burn.’

Several MTLs thought that Airmen used them because they were ‘electronic’, ‘cool lights and stuff, like they can get different colors and it’s like cooler than their phone…and they can look like a dragon.’ They also mentioned that Airmen ‘like to do “smoke tricks” with the devices.’ E-cigarettes were frequently referred to as ‘faddish’, particularly in certain career fields, such as ‘an electronics career field’ (Airman).

### Using e-cigarettes helps with emotion management

Another consistent reason that was identified was emotion management. This included using e-cigarettes to cope with the high stress nature of Technical Training as well as periods of time when they have nothing to do, particularly on weekends and after the duty day (Table 2). One Airmen described the challenging training environment as follows: ‘You ever been in a math class and the question is four plus four, and you know the answer is eight, but they tell you it’s seven? That is exactly how the past three months of our lives have been.’ Another Airmen mentioned how the perception that vaping could relieve stress promoted its use among Airmen in Technical Training. Similarly, several Airmen felt that using e-cigarettes ‘helps you concentrate…it kind of woke me up. It works sometimes better than caffeine.’ Some Airmen mentioned that they just enjoyed the act of smoking, ‘it passes the time and it’s relaxing to me.’

E-cigarette use was also seen as an activity that the Airmen could engage in to manage boredom. A Military Training Instructor mentioned that in Technical Training, there is ‘nothing else to do’ during their free time. Another instructor pointed out that because ‘the majority can’t drink,’ because they are underage, vaping is ‘something else that they do.’

### Vaping is a way of fitting in

Another facilitator of vaping in Technical Training was that it was spread through peer interactions, socially accepted, part of the culture, and the social thing to do (Table 3). One TTI described it as, ‘I think smoking’s the same, but the toys have changed.’ MTLs also mentioned that vaping was spread through ‘peer to peer’ interactions. Supporting this idea, a number of Airmen mentioned borrowing an e-cigarette for the first time in Technical Training from a fellow Airman. Airmen, MTLs and TTIs all discussed how common it was for Airmen to vape in their rooms, even with a roommate that didn’t use tobacco. Several MTLs mentioned that Airmen were not always willing to report to the MTLs when their roommates were vaping in their rooms. Vaping was also seen as part of the culture of Technical Training, and popular among certain career fields. A number of Airmen mentioned how vaping had a social component in Technical Training. One Airmen mentioned that they go to the designated tobacco use areas outside of their dorms for social reasons, while a TTI said Airmen have vaping parties where ‘they see who can blow out the most smoke.’

### Existing tobacco control policies don’t work for vaping

Existing tobacco control policies were seen as ineffective given that e-cigarettes are easy to conceal and use in your room, current designated tobacco use areas were not attractive to e-cigarette users, and the difficulty in enforcing current e-cigarette policies (Table 4). A consistent theme from Airmen, MTLs and TTIs was that Airmen were vaping in their dorm rooms, computer labs, bathrooms and classrooms despite policy restrictions limiting their use during the duty (training) day and restricting tobacco use to designated areas around the military base.

One reason Airmen reported using e-cigarettes outside of the designated tobacco use areas was that they felt they were easy to conceal. Airmen were not concerned about getting caught with them because some e-cigarettes are small and the pod (which contains the nicotine liquid) can easily be removed. Therefore, if they did get caught with one on them they could remove the pod and wouldn’t get in trouble for having it when they weren’t supposed to during their duty day (e.g., when they were in class or training). In fact, some Airmen even mentioned that although they liked the modifiable e-cigarettes more than the smaller rechargeable pod e-cigarettes (e.g., JUUL e-cigarette), they chose to keep ‘stealth’ e-cigarettes with them during the duty day. One Airman even demonstrated how they could easily put a small rechargeable pod e-cigarette in the upper arm pocket of their uniform and use it without anyone knowing what they were doing.

Many Airmen reported vaping in their rooms. Even among those who went to the designated tobacco use areas, many still reported vaping in their rooms. Several Airmen mentioned that leaving their room to go to the designated tobacco use area felt like a hassle because of the heat or it wasn’t worth making a trip all the way outside. Airmen also reported vaping in their dorm rooms was because they didn’t want to be exposed to secondhand smoke at the designated tobacco use areas. Airmen mentioned that when they visited the designated tobacco use areas they ‘got a headache because there were so many different things going on.’ There was also some confusion as to whether the Airmen are allowed to use e-cigarettes in their rooms. One Airman when asked if they were allowed to use e-cigarettes in their dorm rooms said, ‘I have no idea, but I do it.’ While another Airman said, ‘I forget [the rule] all the time.’ Many Airmen felt like the rules were not enforced. However, the MTLs and TTIs expressed how it was hard to catch Airmen in the act of vaping.

## Discussion

The current study sought to identify facilitators of e-cigarette use among Airmen undergoing Technical Training for the purpose of informing future intervention and policy efforts for youth and young adults. While use was initially motivated by factors that have been previously associated with traditional forms of tobacco (e.g., entertainment value, flavor options, and use as an emotion regulation tool), continued use was facilitated by the unique design of e-cigarettes which allowed for users to easily circumvent existing tobacco control regulations.

Airmen’s use of e-cigarettes was motivated by several perceived benefits, such as their entertainment value and use as an emotion regulation tool. Airmen reported using e-cigarettes to help them relax or calm down, or even to help them concentrate. These findings are similar to reasons given for use of other non-cigarette tobacco products, like hookah. Young adults have reported using hookah because of the social aspect, they enjoyed the taste, and smoking hookah produced a calming/relaxation effect [19]. Additionally, Airmen in the sample were drawn to the features of the e-cigarettes, such as lights, flavors and the ability to do smoke tricks. Previous literature has shown that flavors are a primary reason for e-cigarette use among young adults [20-23]. However, in 2020, the Food and Drug Administration (FDA), banned mint- and fruit-flavored e-cigarette liquids except for larger, less discrete tank-based systems or disposable pods. It remains to be seen how this new limitation will impact e-cigarette use among young adults, given that most e-cigarette users purchase flavors other than tobacco [20].

Use of e-cigarettes was reinforced by the unique design of e-cigarettes, affording users with a product that is both discrete and easy to use. Despite explicit policies banning the use of tobacco products during the duty day, many Airmen mentioned that e-cigarettes could easily be concealed and used throughout the day. Previous studies have found that stealth vaporisers, like JUUL, which resemble ordinary devices like USB sticks, are often not recognized by adults, and are therefore popular among youth and young adults [24,25]. Use indoors was also commonplace in our sample, with participants reporting use in bathrooms and their dorms despite explicit rules against use outside of designated tobacco use areas. The nature of using an e-cigarette (e.g., the ability to take a drag every few minutes), promoted their use outside of designated areas; it didn’t make sense to Airmen to go all the way to the designated tobacco use areas for a drag. Additionally, many Airmen avoided the designated tobacco use areas because they did not want to be exposed to the secondhand smoke from burnt tobacco. It may be the case that having one catch-all tobacco use area that encompasses all types of products may not be a viable or acceptable option for e-cigarette users.

In addition, a factor that complicated this situation was the difficulty of catching Airmen using in places or at times that they were not supposed to, making these rules difficult to enforce. School districts around the U.S. are facing similar challenges, and as a result have enacted school-wide flash drive bans, removed the main doors from student bathrooms and installed vapor detectors [24]. In order to combat the increase in e-cigarette use among Airmen in prohibited areas, the Air Force may need to consider implementing similar measures.

There was also a pervasive theme related to the belief that e-cigarettes were not harmful, and there was no concern for e-cigarette secondhand smoke as evidenced by use in dorms with non-smoking roommates. While e-cigarette secondhand smoke does not contain the same toxicants as a traditional cigarette, there may be other reasons for health concern. Studies have shown that e-cigarette users exhale some of the mainstream vapor, which exposes bystanders to harmful constituents including heavy metals, nicotine, ultrafine particulates, volatile organic compounds and other toxicants [26-28]. Another reason for using in places where e-cigarettes were prohibited was confusion about whether e-cigarettes were considered tobacco. This limited awareness of tobacco policies is similar to a study conducted with 14 colleges and universities, which found that most students did not know if their school included e-cigarettes in their tobacco policies, or if there were e-cigarette use policies despite the fact that most students also reported being supportive of having such a policy [29]. These findings suggest that there is a need for specific policies related to e-cigarettes, and concerted efforts to raise awareness about these policies may help clarify the inclusion of e-cigarettes and limit e-cigarette use. However, additional research is needed to examine the role that awareness of policies has on e-cigarette use behaviors.

An additional consideration is the implementation of the new federal Tobacco 21 law, which prohibits the sale of tobacco to anyone under the age of 21 even if they are military personnel [30]. Almost half of Airmen are under the age of 21 and thus are no longer able to buy their own e-cigarette products [20,31]. A common facilitator at the time of the focus groups (conducted prior to the implementation of the Tobacco 21 law) was that e-cigarette use was something Airmen could legally do given there were not a lot of ways Airmen could spend their free time. With this new legislation, Airmen will no longer be allowed to purchase tobacco if they are under 21, but will still be allowed to use tobacco [30]. This could lead some Airmen that are of age to purchase tobacco products for underage Airmen, which, if enforced similarly to the rules around alcohol use in the military, would be classified as ‘contributing to a minor’ and be a punishable offense leading to a delay in training or even early discharge. Given that the Air Force already loses $18 million annually on excess training costs associated with smoking [32], it is likely that this number will increase under the new legislation. Additional research is needed to determine if this new regulation is effective at reducing e-cigarette use among youth and young adults, or if they simply get these products elsewhere, such as from a friend or roommate, which may require the Department of Defense to take additional steps to strengthen their approach to tobacco control. For instance, in the United Kingdom, plain packaging and a smoking ban in cars with minors was recently enacted, while other European countries are striving to reduce the prevalence of youth smoking to under 5% in the next 15-20 years [33,34]. To achieve this goal, countries like Ireland, Scotland, Finland, France and the Netherlands have all adopted more stringent tobacco control policies, such as plain packaging, point-of-sale display bans and smoke-free playgrounds [33,34]. In 2016, the Department of Defense put out a policy memo with a similar goal to reduce tobacco use and secondhand smoke exposure by restricting tobacco use to designated areas on base, creating multi-unit smoke-free military housing, and increasing the price of tobacco [35]. It is yet to be seen how effective these policies will be at reducing tobacco use among military personnel.

## Strengths and Limitations

The current study was not limited to e-cigarette product use, and therefore we did not assess all potential reasons for e-cigarette use, or if specific themes were related to actual e-cigarette use behaviors. However, the current study provides insights related to perceptions and beliefs about e-cigarette use among a large and diverse sample of young adults and can inform future e-cigarette policies and interventions.

## Conclusion

Results highlight how the unique design of e-cigarettes coupled with perceptions that e-cigarettes are less harmful and socially accepted promote circumventing existing tobacco control regulations. In order to effectively reduce the growing prevalence of e-cigarette use, existing policies and interventions will need to take a systems approach, in which multiple levels of influence (individual, community and environment) are considered.

## Figures and Tables

**Table 1:** Theme: No reasons not to use e-cigarettes.

Theme	Representative Quotes
*Positive attributes*	‘It tastes good. Compared to like, regular cigarettes, it tastes way better.’ (Airman) This smells like bubble gum so I’m just gonna do it.’ (MTL) ‘It doesn’t smell and you can get the same nicotine, but it has got this flavor and it doesn’t taste like a normal cigarette. You can get chocolate or strawberry or bubble gum.’ (TTI)
*Not perceived as harmful*	‘Vaping is hardly tobacco’ (Airman) ‘Every single person that I've talked to about vaping says, "Oh, it's not bad. It doesn't have any negative affects whatsoever."’ (Airman) ‘Why am I gonna go out to the smoke pit, it’s not like I’m smoking a cigarette that’s gonna stink up the room.’ (Airman)
*Cool gadgets*	Airmen use them because they are ‘electronic’, ‘cool lights and stuff, like they can get different colors and it’s like cooler than their phone… and they can look like a dragon.’ (MTL) Airmen ‘like to do “smoke tricks” with the devices.’ (MTL) “‘Wow, that looks like a cool new gadget to have.” Especially if you are a tech person, they are just neat little things to have.’ (MTL) ‘…our class has had conversations with them [TTIs] about how their vapes… they can make their own, make your own modifications to it and stuff, especially in an electronics career field, it's really big’. (Airman)
*Easy to use*	‘You just hit it once and you are done…Instead of lighting up a cigarette and letting it burn.’ (TTI)

**Note:** MTL: Military Training Leader; TTI: Technical Training Instructor

**Table 2:** Theme: Using e-cigarettes helps with emotion management.

Theme	Representative Quotes
*High stress nature of Technical Training*	‘You ever been in a math class and the question is four plus four, and you know the answer is eight, but they tell you it's seven? That is exactly how the past three months of our lives have been.’ (Airman) ‘Helps you concentrate…it kind of woke me up. It works sometimes better than caffeine.’ (Airman) ‘It passes the time and it's relaxing to me.’ (Airman)
*Nothing else to do*	‘Nothing else to do’ (MTL) ‘[Because] the majority can't drink,’ [they are underage, vaping is] ‘something else that they do.’ (TTI) ‘Because your duty day [scheduled training and activities] ends or on the weekends, [there is] not a lot going on.’(Airman)

**Note:** MTL: Military Training Leader; TTI: Technical Training Instructor.

**Table 3:** Theme: Vaping is a way of fitting in.

Theme	Representative Quotes
*Spread through peer interactions*	‘Yeah, I borrowed one of theirs and just hit it a few times and I was like, "You know what, let's go to the vape shop."’ (Airman) ‘Say you go to a study session and one person there has a vape and he's like, "It helps me calm down," the next person uses it and then the whole class tries it. Maybe it affects two or three of them and they all go buy one.’ (Airman)
*Socially accepted*	‘I don’t know if my roommate cares, but she hasn't said anything, so I just walk around the room [vaping]…If my roommate cared, she would've said something by now.’ (Airman)
*Part of the culture*	‘Everyone that I heard coming out of tech school was like, "Oh, yeah, when you go into tech school, you'll come out vaping. It's just what everyone does."’ (Airman) ‘Everybody’s doing it. And just like oh, your friend’s doing it here. They’re easily peer pressured here… You have good Airmen that just don’t want to be alone anymore.’ (MTL)
*The social thing to do*	‘I went out there [designated tobacco use area] because we were just hanging out; I didn't go there to smoke. I brought my Juul but I wasn't going there just to do that.’ (Airman)

**Note:** MTL: Military Training Leader; TTI: Technical Training Instructor.

**Table 4:** Theme: Existing tobacco control policies don’t work for vaping.

Theme	Representative Quotes
*E-cigarettes are easy to conceal*	‘People [Airmen] will do it in the bathroom during the duty day. People [Airmen] will do it in classrooms during the duty day. It's easy.’ (Airman) ‘Have a stealth [concealable e-cigarette] on me usually, all the time.’ (Airman) ‘Yeah, you can put it in here and then you just [demonstrates using an e-cigarette from a device without removing it from their pocket]…’ (Airman)
*E-cigarettes are easy to use in your room*	‘You're only supposed to do it in the designated smoke area, but people vape [use e-cigarettes] in their rooms all the time.’ (Airman) “Sometimes, I forget. I do it so often, I'll forget when my door's open [to my dorm room] that I'm not supposed to be doing it.” (Airman) ‘Even if you go to the smoke pit with your e-cig, you're still probably using it in your room when you're not at the smoke pit.’ (Airman) ‘I don't even think about going to the smoke area. It's literally smoking for five seconds.’ (Airman)
*Designated tobacco use areas are not attractive to e-cigarette users*	‘I hate going to the smoke pit [designated tobacco use area] because they're sitting out there smoking cigarettes’. (Airman) ‘It’s easier to get my nicotine buzz from something [e-cigarette] up in my room.’ (Airman) ‘You go up there [designated tobacco use area] and have this guy puffing banana flavored mist this way, and cigarette smoke that way’ (Airman). ‘It seems like a big to-do to go and leave your room.’ (Airman) ‘Why would I sit outside and smell up my breath and my clothes if I can just get twice the nicotine and more of the buzz just chilling in my room?’ (Airman)
*Difficult to enforce current e-cigarette policies*	‘Not everyone [i.e., potential rule enforcers] knows what it looks like or what it smells like.’ (Airman) ‘It won't turn on if you don't have the pod in. You can just be like, "Yeah, I have this but there's no contraband in it."’ (Airman) ‘Nobody gets caught.’ (Airman) ‘Yeah, and like I said, unless you catch them actually doing it, with a vape [e-cigarette], it's hard to [prove it]… you have to prove it.’ (MTL)

**Note:** MTL: Military Training Leader; TTI: Technical Training Instructor.

## References

[1] Creamer MR, Wang TW, Babb S, Cullen KA, Day H, (2019) Tobacco Product Use and Cessation Indicators Among Adults - United States, 2018. MMWR 68: 1013–1019. 10.15585/mmwr.mm6845a2 PMC6855510 31725711

[2] Agaku IT, King BA, Husten CG, (2014) Tobacco product use among adults--United States, 2012-2013. MMWR Morb Mortal Wkly Rep 63: 542–547. 24964880 PMC5779380

[3] Little MA, Klesges R, Wang XQ, Fahey MC, Ebbert JO, 2019 Are E-cigarettes a Gateway Product in Young Adults? A Longitudinal Investigation in the United States Military. Presentation given at the National Institutes of Health Tobacco Regulatory Science Meeting in Bethesda, MD 21–23.

[4] Goniewicz ML, Lingas EO, Hajek P (2013) Patterns of electronic cigarette use and user beliefs about their safety and benefits: an internet survey. Drug Alcohol Rev 32: 133–140. 22994631 10.1111/j.1465-3362.2012.00512.x PMC3530631

[5] Ghosh A, Coakley RD, Ghio AJ, Muhlebach MS, Esther CR Jr, (2019) Chronic E-cigarette use increases neutrophil elastase and matrix metalloprotease levels in the lung. Am J Respir Crit Care Med 200: 1392–1401. 31390877 10.1164/rccm.201903-0615OC PMC6884043

[6] Madison MC, Landers CT, Bon-Hee G, Cheng-Yen C, Hui-Ying T, (2020) Electronic cigarettes disrupt lung lipid homeostasis and innate immunity independent of nicotine. J Clin Invest 129: 4290–4304. 10.1172/JCI128531 PMC6763255 31483291

[7] Chung S, Baumlin N, Dennis JS, Moore R, Salathe SF, (2019) Electronic Cigarette Vapor with Nicotine Causes Airway Mucociliary Dysfunction Preferentially via TRPA1 Receptors. Am J Respir Crit Care Med 200: 1134–1145. 31170808 10.1164/rccm.201811-2087OC PMC6888648

[8] Robbins A, Chao S, Coil G, Fonseca V (2000) Costs of smoking among active duty U.S. Air Force personnel— United States, 1997. MMWR 49: 441–445. 10843504

[9] Klesges RC, Haddock CK, Lando H, Talcott GW (1999) Efficacy of forced smoking cessation and an adjunctive behavioral treatment on long-term smoking rates. J Consult Clin Psychol 67: 952–958. 10596516 10.1037//0022-006x.67.6.952

[10] Miller MGRI. Tobacco Free Living. In: Force SotA, ed. Air Force Instruction 48-104. Vol Air Force Policy Directive (AFPD) 48–1.

[11] Richardson L, Hemsing N, Greaves L, Assanand S, Allen P, (2009) Preventing smoking in young people: a systematic review of the impact of access interventions. Int J Environ Res Public Health 6:1485–1514. 19440530 10.3390/ijerph6041485 PMC2681197

[12] Dunkle A, Kalpinski R, Ebbert J, Talcott W, Klesges R, (2018) Predicting smokeless tobacco initiation and re-initiation in the United States Air Force. Addict Behav Rep 9: 100142. 31193918 10.1016/j.abrep.2018.11.001 PMC6544562

[13] Little MA, Ebbert JO, Krukowski RA, Halbert JP, Kalpinski R, (2019) Predicting cigarette initiation and reinitiation among active duty United States Air Force recruits. Subst Abus 40: 340–343. 30883297 10.1080/08897077.2019.1577678 PMC6751016

[14] Lopez CT (2019) Military Exchanges Extinguish Vape Sales. In. U.S. Dep of Defense.

[15] Fahey MC, Talcott GW, McMurry T, Klesges RC, Tubman D, (2020) When, How, & Where Tobacco Initiation and Relapse Occur During U.S. Air Force Technical Training. Mil Med 185: e609–e615. 32060547 10.1093/milmed/usaa016 PMC7282443

[16] Fahey MC, Krukowski RA, Talcott GW, Little MA (2020) JUUL targets military personnel and veterans. Tobacco Control. 10.1136/tobaccocontrol-2019-055377 PMC8101685 32398271

[17] Haddock CK, O’Byrne KK, Klesges RC, Talcott W, Lando H, (2000) Relapse to smoking after basic military training in the U.S. Air Force. Mil Med 165: 884–888. 11143440

[18] National Center for Chronic Disease Prevention and Health Promotion (US) Office on Smoking and Health. E-Cigarette Use Among Youth and Young Adults: A Report of the Surgeon General. Atlanta (GA): Centers for Disease Control and Prevention 2016. 30869850

[19] Nicksic NE, Ly C, Loukas A, Perry CL (2018) Hookah Use and Perceptions among Young Adult Hookah Users. J Addict Behav Ther Rehabil 7. 10.4172/2324-9005.1000178 PMC6831090 31692999

[20] Landry RL, Groom AL, Vu TT, Stokes AC, Berry KM, (2019) The role of flavors in vaping initiation and satisfaction among U.S. adults. Addict Behav 99: 106077. 31437770 10.1016/j.addbeh.2019.106077 PMC6903386

[21] Goldenson NI, Leventhal AM, Simpson KA, Barrington-Trimis JL (2019) A Review of the Use and Appeal of Flavored Electronic Cigarettes. Curr Addict Rep 6: 98–113. 31453046 10.1007/s40429-019-00244-4 PMC6709993

[22] Rostron BL, Cheng Y-C, Gardner LD, Ambrose BK (2020) Prevalence and Reasons for Use of Flavored Cigars and ENDS among US Youth and Adults: Estimates from Wave 4 of the PATH Study, 2016-2017. Am J Health Behav 44: 76–81. 31783934 10.5993/AJHB.44.1.8 PMC6918456

[23] Villanti AC, Johnson AL, Glasser AM, Rose SW, Ambrose BK, (2019) Association of Flavored Tobacco Use With Tobacco Initiation and Subsequent Use Among US Youth and Adults, 2013-2015. JAMA Netw Open 2:e1913804. 31642927 10.1001/jamanetworkopen.2019.13804 PMC6820032

[24] Ramamurthi D, Chau C, Jackler RK(2019) JUUL and other stealth vaporisers: hiding the habit from parents and teachers. Tobacco Control 28: 610–616. 10.1136/tobaccocontrol-2018-054455 30219794

[25] Peters RJ, Meshack A, Lin M-T, Hill M, Abughosh S (2013) The Social Norms and Beliefs of Teenage Male Electronic Cigarette Use. Journal of Ethnicity in Substance Abuse 12: 300–307. 24215223 10.1080/15332640.2013.819310

[26] Czogala J, Goniewicz ML, Fidelus B, Zielinska-Danch W, Travers MJ, (2014) Secondhand exposure to vapors from electronic cigarettes. Nicotine Tob Res 16: 655–662. 24336346 10.1093/ntr/ntt203 PMC4565991

[27] U.S. Department of Health and Human Services (2016) E-Cigarette Use Among Youth and Young Adults. A Report of the Surgeon General. Atlanta, GA: U.S. Department of Health and Human Services, Centers for Disease Control and Prevention, National Center for Chronic Disease Prevention and Health Promotion, Office on Smoking and Health 2016.

[28] Schripp T, Markewitz D, Uhde E, Salthammer T (2013) Does e-cigarette consumption cause passive vaping? Indoor Air 23: 25–31. 22672560 10.1111/j.1600-0668.2012.00792.x

[29] Brown EM, Henes AL, Olson LT (2016) E-Cigarette Policies on College Campuses: Student Use Behaviors, Awareness, and Policy Support. J Community Health 41: 1110–1115. 27655585 10.1007/s10900-016-0262-y

[30] U.S. Food & Drug Administration (2020) Tobacco 21.

[31] Little MA, Derefinko KJ, Bursac Z, Ebbert JO, Colvin L, (2015) Prevalence and Correlates of Tobacco and Nicotine Containing Product Use in a Sample of United States Air Force Trainees. Nicotine Tob Res 18: 416–423. 25895952 10.1093/ntr/ntv090 PMC4854492

[32] Klesges RC, Haddock CK, Chang CF, Talcott GW, Lando HA (2001) The association of smoking and the cost of military training. Tob Control 10: 43–47. 11226360 10.1136/tc.10.1.43 PMC1764000

[33] Nuyts PAW, Kuipers MAG, Willemsen MC, Kunst AE (2019) An Increase in the Tobacco Age-of-Sale to 21: For Debate in Europe. Nicotine Tob Res 22: 1247–1249. 10.1093/ntr/ntz135 PMC7291794 31408159

[34] Organization WHO (2017) Tobacco-free generations: protecting children from tobacco in the WHO European Region. WHO Europe 1–36.

[35] Carter A (2016) Policy Memorandum 16-001, Department of Defense Tobacco Policy. In: Secretary of Defense, ed. 16-001. Washington, DC: Secretary of Defense.

